# Validation of virtual reality orbitometry bridges digital and physical worlds

**DOI:** 10.1038/s41598-020-68867-6

**Published:** 2020-07-16

**Authors:** Peter M. Maloca, Balázs Faludi, Marek Zelechowski, Christoph Jud, Theo Vollmar, Sibylle Hug, Philipp L. Müller, Emanuel Ramos de Carvalho, Javier Zarranz-Ventura, Michael Reich, Clemens Lange, Catherine Egan, Adnan Tufail, Pascal W. Hasler, Hendrik P. N. Scholl, Philippe C. Cattin

**Affiliations:** 1 Institute of Molecular and Clinical Ophthalmology Basel (IOB), 4031 Basel, Switzerland; 2 OCTlab, Department of Ophthalmology, University Hospital Basel, 4031 Basel, Switzerland; 3 Department of Ophthalmology, University of Basel, 4031 Basel, Switzerland; 4 Moorfields Eye Hospital NHS Foundation Trust, London, EC1V 2PD UK; 5 Center for Medical Image Analysis & Navigation, University of Basel, 4031 Basel, Switzerland; 6 MRZ Medical Radiology Center, 6004 Lucerne, Switzerland; 7 Institut Clínic d’Oftalmologia, Hospital Clínic de Barcelona, 08036 Barcelona, Spain; 8 Faculty of Medicine, Eye Center, Albert-Ludwigs University Freiburg, 79085 Freiburg, Germany; 9 Wilmer Eye Institute, Johns Hopkins University, Baltimore, 21287 USA

**Keywords:** Imaging, Software, Structure determination

## Abstract

Clinical science and medical imaging technology are traditionally displayed in two dimensions (2D) on a computer monitor. In contrast, three-dimensional (3D) virtual reality (VR) expands the realm of 2D image visualization, enabling an immersive VR experience with unhindered spatial interaction by the user. Thus far, analysis of data extracted from VR applications was mainly qualitative. In this study, we enhance VR and provide evidence for quantitative VR research by validating digital VR display of computed tomography (CT) data of the orbit. Volumetric CT data were transferred and rendered into a VR environment. Subsequently, seven graders performed repeated and blinded diameter measurements. The intergrader variability of the measurements in VR was much lower compared to measurements in the physical world and measurements were reasonably consistent with their corresponding elements in the real context. The overall VR measurements were 5.49% higher. As such, this study attests the ability of VR to provide similar quantitative data alongside the added benefit of VR interfaces. VR entails a lot of potential for the future research in ophthalmology and beyond in any scientific field that uses three-dimensional data.

## Introduction

Orbital tumors can either be primary, arising from structures within the orbit, or secondary, due to metastatic spread of a primary tumor elsewhere^1–3^. A myriad of clinical findings suggest the presence of an orbital mass, namely proptosis, diplopia, pain, conjunctival congestion and varying degrees of visual loss^4,5^.

Diagnosis relies on adequate clinical examination and orbital imaging like magnetic resonance imaging (MRI) and/or computed tomography (CT)^6^. Despite the great progress in medical imaging, the diagnosis of orbital lesions based on neuroimaging features may lack characteristic imaging features. Often, the diagnosis of specific orbital disorders such as Graves’ disease associated orbitopathy, orbital inflammation, orbital lymphoma, lacrimal gland epithelial tumor, metastatic carcinoma, and vascular orbital lesions is ambiguous^7–10^. Confirmation of the diagnosis frequently relies on a biopsy which can be technically challenging and prone to complications due to the dense arrangement of tissue or inaccessibility of many of these lesions^11^.

Visualization of orbital lesions is still based on pre-surgery image assessment of two-dimensional (2D) data visualization on a computer monitor or on intraoperative exploration^12,13^. There are increasing possibilities for presentation and interaction with virtual reality^14^. In this context, virtual reality (VR) has recently been optimized as an enhanced medical image display method and showed to safeguard visual comfort^15,16^.

The main objective of this study was to extend current medical image display and validate the level of spatial precision in orbitometry of CT data, representing the physical world, compared to precision in three-dimensional (3D) virtual reality for the first time. This is especially important when new ways are explored to use emerging and transforming digital media to virtually guide a surgeon for saving and improving lives.

## Results

Three graders measured in the physical world, while four graders used the VR device (one female, 14% and six males, 86%). The graders were 42 years old on average (range from 27 to 68 years). Four graders were right-handed (57%) and three left-handed (53%), present in both groups. The physical domain experts comprised two radiologists with an average work experience of 18.33 years, and one ophthalmologist with a work experience of 23 years. The virtual world experts had an average VR work experience of 3 years.

**Figure 1:**
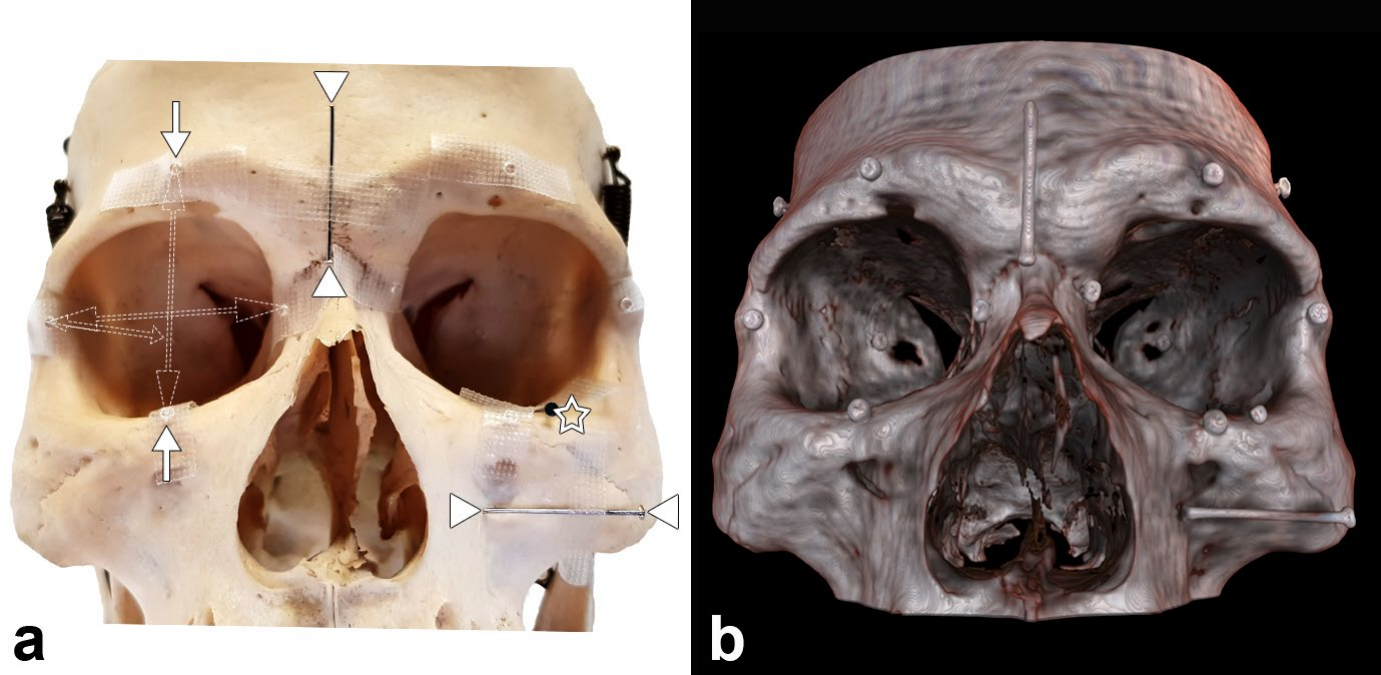
Diameter measurements of physical world compared to digital VR world of the human orbit. Two different groups of measuring landmarks were placed on a physical skull. (**a**) For each orbit, six small metal beads were placed in the three main axes, defining three different diameters (for better overview, these were illustrated only on the right orbit; single arrows representing the longitudinal beads, double headed arrows indicate the measured longitudinal and frontal diameter measured from the outside border; short double arrow indicates the oblique and lateral diameter inside the orbit). In addition, three metal pins (arrow head) were attached in the aforementioned axes on the skull near the orbit, whereby only the head of the third nail (asterisk) is visible from outside. VR imaging (**b**) of the identical skull from (**a**) displays the corresponding landmarks. The osseous structures are rendered realistically in VR, whereas the metal particles appeared somewhat enlarged. The metallic objects are relatively sharp-edged in VR. No image degradation has been noted, indicating that the used landmarks and the scan protocol parameters during CT acquisition were appropriate. Abbreviations: computed tomography (CT); virtual reality (VR). The figure was created using Adobe Photoshop 2020 (Adobe Inc., San Jose, US, licence 65230035) and image b was obtained from the described software SpectoVR (version 3.1.0, Diffuse ltd., Heimberg, Switzerland, https://www.diffuse.ch).

Nine different diameters were measured repeatedly, three times each (Fig. 1).

This resulted in a total of 189 diameter measurements (81 in the physical world and 108 in the virtual reality). The results of the statistical analysis are summarized in Tables 1 and 2.

**Table 1:** Mean diameter measurements using two different kinds of landmarks.

Location	Physical world	Virtual world	Difference
**Beads**			
1	41.0	42.91	1.92
2	41.8	44.32	2.51
3	39.6	43.67	4.05
4	41.1	43.30	2.22
5	28.2	30.12	1.90
6	32.5	35.06	2.52
Min	28.22	30.12	1.90
Max	41.81	44.32	4.05
Mean	37.38	39.90	2.52
**Pins**			
7	25.289	26.682	1.39
8	24.661	25.806	1.15
9	25.078	25.73	0.65
Min	24.66	25.73	1.07
Max	25.29	26.68	1.39
Mean	25.01	26.07	1.06

Length in millimeters (mm).

The beads landmarks (#1–6) provided a larger deviation compared to the relative stiff metal pins (#7–9).

**Table 2:** Intraclass correlation coefficient of orbital diameter measurements for the physical grader (a) and the VR grader (b). A value between 0.75 and 1.00 is considered good.

Model type unit	Type	Unit	ICC	95%
(a)				
Twoway	Agreement	Average	ICC(A,3) = 0.995	0.978 < ICC < 0.999
Twoway	Agreement	Single	ICC(A,1) = 0.984	0.936 < ICC < 0.996
Twoway	Consistency	Average	ICC(C,3) = 0.997	0.989 < ICC < 0.999
Twoway	Consistency	Single	ICC(C,1) = 0.990	0.968 < ICC < 0.997
(b)				
Twoway	Agreement	Average	ICC(A,4) = 1.000	1.000 < ICC < 1.000
Twoway	Agreement	Single	ICC(A,1) = 1.000	0.999 < ICC < 1.000
Twoway	Consistency	Average	ICC(C,4) = 1.000	1.000 < ICC < 1.000
Twoway	Consistency	Single	ICC(C,1) = 1.000	0.999 < ICC < 1.000

**Figure 2:**
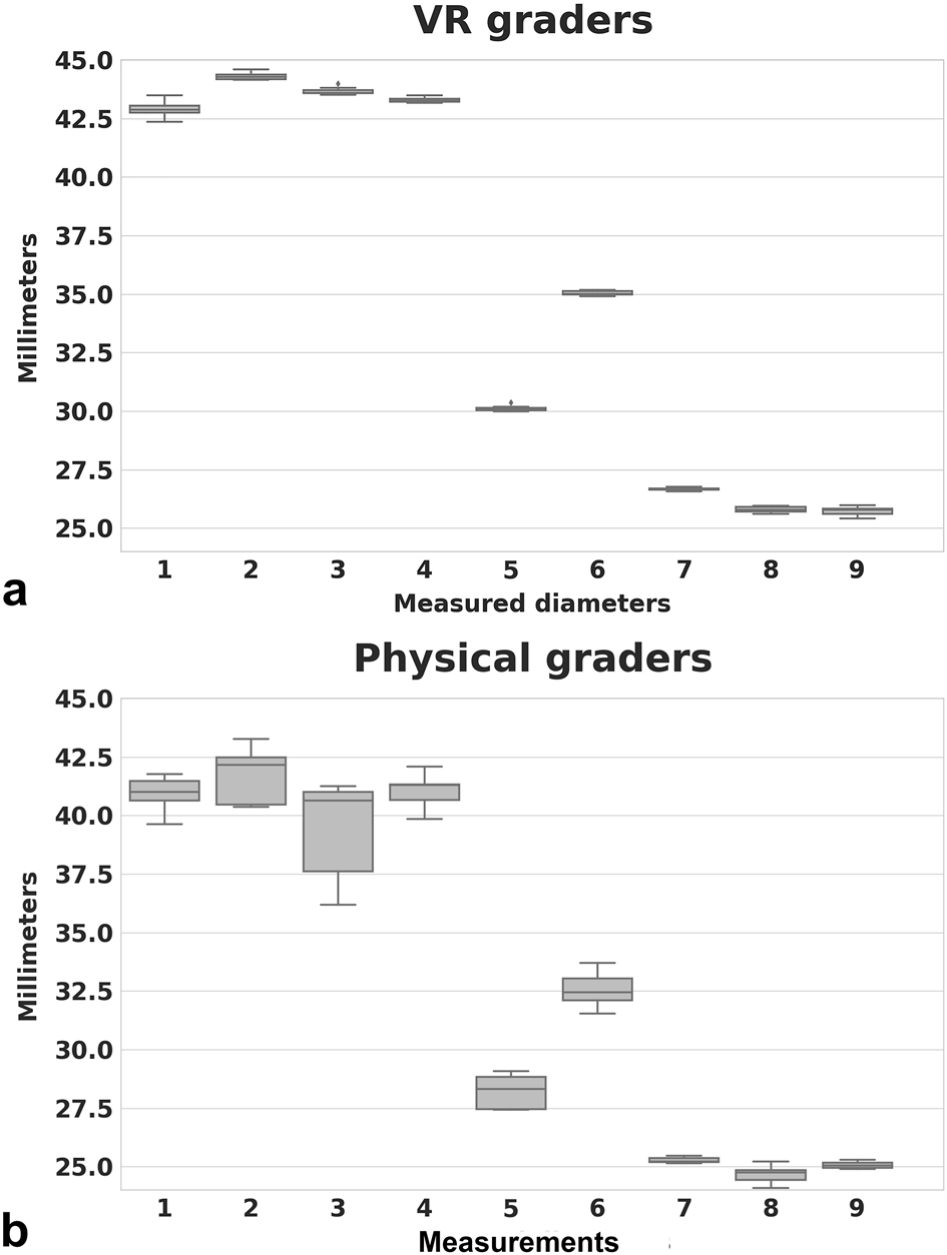
Results of physical diameter measurements compared to digital VR world measurements. In both worlds, it was possible to measure all diameters. In the physical world (**a**), using a caliper, the deviation appeared higher when compared to VR. The oblique diameter (location # 3) was not optimally measurable. In contrast, the pins that were placed in relatively easily accessible and visually controllable locations showed significantly less variation. In the VR (**b**), the results are consistent, irrespective of the location.

Virtual reality measurements (Fig. 2) showed 6.74% and 4.25% higher values for beads and nail landmarks, respectively. Overall, the VR readings were on average 5.49% higher.

The reproducibility of the method was good (Intraclass correlation coefficient, ICC, 95% confidence interval [CI] 0.984 to 1.0, p < 0.01).

The physical and virtual measurements differ statistically significantly (p < 0.01) and so also their variances (p < 0.01). The effect size between the physical and virtual measurements 2–6 was relatively large (d > 1.2) while for 1, 7 and 8 it was medium (d > 0.5) and for 9 small (d > 0.01).

**Figure 3:**
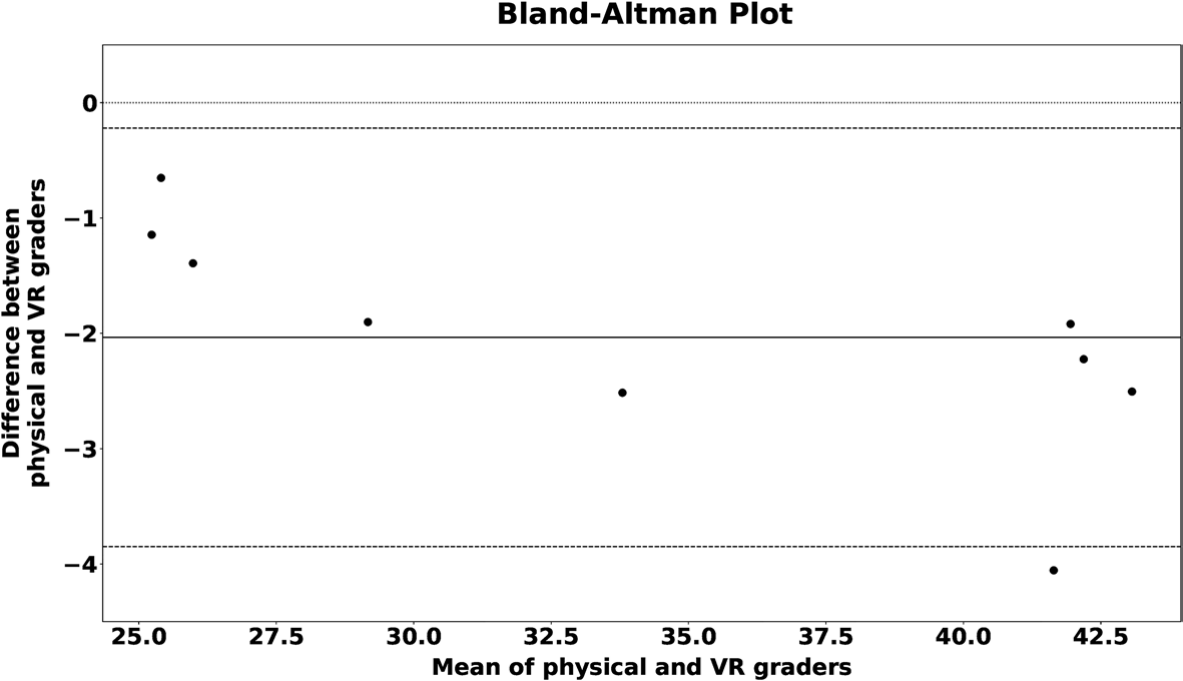
This Bland–Altman plot visualizes the agreement between the physical and virtual measuring method. An average difference of 2 mm (solid line) is observed. The differences lie within the 95% confidence interval (dashed lines) with one exception.

**Figure 4:**
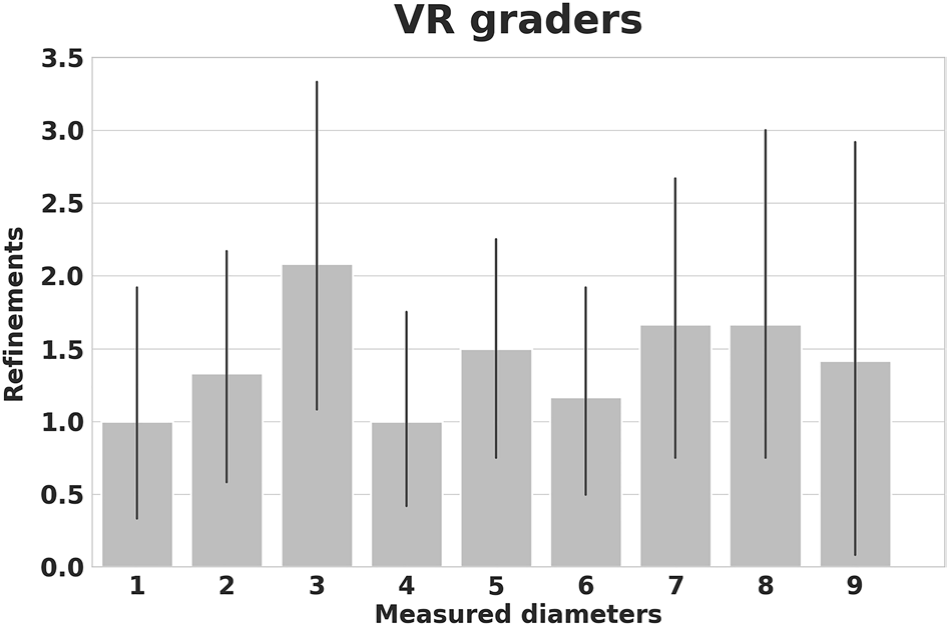
Refinement bar plots of diameter measurements in virtual reality (VR). Because the VR users were always digitally tracked, it was possible to record and display their performance instantly: An additional parameter is offered by VR by determining how often a measurement had been corrected before it had been saved. The mean overall correction of all graders was 1.5. This is not quantifiable in the physical world and may serve as a novel benchmark regarding the performance of a VR user.

Overall, a good agreement between graders within the physical or the VR world was found respectively as indicated by a William’s index close to 1.0 for all subjects in both worlds. While all VR measurements were reliable, a significant location-dependent inter-reader variability of measurements was found in the physical world (Figs. 2, 3). Here, the best measurement results were obtained for the more easily accessible locations, while the reliability of the oblique diameter measurement inside the orbit was inferior. As a further dimension of confidence in the measurement, the VR setting allowed for recording how often a measurement had been corrected before it had been saved. The number of refinements performed by the VR graders was low with a mean of 1.5 (Fig. 4).

## Discussion

Immersive technologies (ITs), namely digital realities such as virtual reality and augmented reality (VR and AR) will profoundly modify how we interact with scientific content and beyond. Researcher and clinicians will move from observing their data on rectilinear display devices to immerse themselves at the center of an ever-expanding virtual world that provides experiences and an understanding of data that has never been seen before. Despite the fact that a vast majority of scientist still never have used VR for their research it seems likely that these technologies will become increasingly integrated as a data platform in research. While most ITs are still under evaluation especially as pure qualitative analysis tools, there is an emerging need for adding measureable and objective data to digital scientific content.

Therefore, to develop and deploy VR with a robust, distinct utility for the scientist, the present study successfully compared completely independent graders for the validation of diameter measurements in the physical and digital VR world. It was shown that VR can be used not only as an emerging method for qualitative image visualization of medical image data^17^, but that quantitative diameter measurements in VR were valid, highly reliable, repeatable and more uniform when compared to physical world measurements^18^.

While of course it is already standard to perform accurate size measurements of anatomical structures on a conventional 2D screen using medical imaging data, to the best of our knowledge, this is the first study presenting the validation of orbital VR diameter measurements. The obtained results appear equivalent to similar studies in other medical subspecialties using a VR environment, photogrammetry or computed tomography (CT)^19–21^.

This study draws attention to the fact that both worlds express some differences in the spatial representation of CT image data. This result is all the more important as more and more studies are presenting simulators for training purposes^22–24^. These differences must be taken into account in the future, as instruments may be rendered incorrectly and implants may be placed in the wrong position, particularly if surgery is required deep inside the orbit, which is difficult to access in the physical world. In these locations, our data suggests the biggest advantage of the VR system, as the VR-based measurement were consistently more reliable.

The slightly increased VR values could be caused by (I) the metal material used for the marking, which might have led to CT blooming artefact signals^25^, or (II) the transfer function used in the VR imaging. As the typical radiating metal CT artefacts^26^ were not detectable in the VR rendering, the metal landmarks seemed unlikely as a cause. Nevertheless, different materials should be tested for their properties regarding CT transmissibility and visualization in VR in future studies. This might also help to define a uniform threshold in order to improve the transfer function used in the VR.

There was a noticeable difference in the measurements between metal beans and the pins, which were rigid in themselves. This finding can be important for upcoming VR orbital measurements or intraoperative navigation performed on tantalum clips used in the course of ocular melanoma treatment where comparable measurement deviations have been described^27^. Nevertheless, these deviations have to be put into perspective since a common hand tremor of 0.2–0.5 mm was also measured despite utilization of tremor reducing surgical instruments^28^, but can be improved with training and optical tracking^29^.

The VR-system revealed more homogeneous measurement that may be traced back to the absence of physical limits that may hinder the grading. The grader can move his instrument freely in space and thus set his measuring marks more precisely. The high confidence in the measurement was indirectly measured and shown by the low rate of refinements which was recorded by this novel VR tool (Fig. 3). This refinement application can be helpful to track the learning curve of a surgeon and compare it with others with regard to differing skill levels in measurement accuracy and refinement. Compared to our system, other VR-systems showed much higher inter-reader variations^30,31^. In these previous studies, the study subjects were not aware of their reduced precision performance. We can only hypothesize the reason for these different results. The higher resolution and frame rate of our displays could have played an important role in the improvement of our measurements.

It is beyond doubt that the navigation of complex 3D data will be a central key for many novel scientific and clinical applications. More VR features were shown to be helpful during this study. Firstly, the 3D model can be magnified to heighten perception of volumetric details. Thus, critical details can be instantly made more accessible. In this context, it was already reported that a higher microscope magnification was useful and resulted in a reduced variability of suture placement on synthetical vascular prostheses^32^.

Secondly, measurements can also be recorded as a stereoscopic video clip for training purposes or patient engagement in pre-surgery decision-making. These enhancements are not possible in the physical world.

A possible limitation of the method could be caused by the relatively small number of graders. It would be possible that more and completely inexperienced graders would have contributed to larger measurement deviations. However, the obtained results showed quite uniform variations within the method, hence it can be assumed that the conclusion still holds true. In times of the COVID-19 pandemic an extension of the study is very difficult as the gathering of several people has been prohibited by Swiss law in order to prevent the virus from spreading. Currently the VR equipment to be shared by several graders indicate a source of increased risk for dissemination of the virus. In addition, the virtual reality glasses cannot be easily cleaned with sterilizing agents because of the fragile optics/soft pads, electronics etc. However, as a result of the massive disruption caused by the COVID 19 pandemic, the research community has become painfully aware that many scientists have faced great difficulties in sharing their data and discoveries with their peers. In this context, virtual reality as a data platform has a great potential to help overcome current barriers and to bridge gaps. Future studies should further evaluate this circumstance. One weakness of this study was the fact that different styles of calipers were not compared with each other. The measurement uncertainty of a caliper could be greater than the resolution of the display, especially if an analogue nonius makes it difficult to read smaller values. The measuring error of a commercially available caliper could hamper the measurements attained. Nevertheless, the caliper used is justifiable because a reliable reading of 0.01 mm was published by the manufacturer^33^. To further reduce errors and facilitate reading, a caliper was used that displayed the measured values digitally and all readings were blinded during measurements to not bias the reading. Nevertheless, the values within the method were accurate and reproducible. Another limit of the digital measurements can also be caused by the tracking capability of the VR controllers held directly in the hands of the user and the precision of it^34^. In order to verify this, further investigations are necessary with different VR setups (tracker positions) and different products. Furthermore, currently there was a lack of a haptic feedback in the VR system that could lead to some impairment of the hand–eye coordination during VR which can reduce depth perception^35,36^. Improving the haptic feedback might lead to even improved reliability of VR-based measures in future. In this preliminary study, only claims for orbitary scenarios were raised and similar research alignment tasks have resulted in comparable performance^37^. Nevertheless, it can be mentioned that the discussed VR application only constitutes a platform for any kind of clinical or research 3D data. Therefore, investigations have already been initiated to evaluate a generalizability to other medical subspecialties, which can be useful for abdominal CT exploration, especially for instance for non-invasive clinical virtual colonoscopy (VC) procedures^38^. In a different and complex domain, the assessment of congenital heart diseases, a successful integration of the VR method mentioned above, combined with 3D print of the cardiac atria, was carried out^39^.

In summary, the orbit and possibly all other three-dimensional spaces can be non-invasively and digitally visualized and measured in close-to-reality conditions and investigated with a high precision using a VR image display method that measured what it purports to measure. An objective diameter measurement can be attained to quantify the dimensions of the orbit and improve spatial awareness, diagnosis, monitoring and pre-surgical planning.

## Methods

### Subjects

A single naturally dried skull was used for diameter measurements of the orbit. The skull was acquired from the Anatomical Institute Fribourg, Switzerland, in 1971. There were no records found regarding the name, gender or genealogical history of the skull. That is why the patient data was anonymous. It was not possible to obtain an informed consent. Research with anonymized data does not fall within the scope of the Swiss Human Research Act and does not need approval of the local ethics committee. The study was performed in accordance with the declaration of Helsinki and in compliance with data-protection regulations.

The diameter measurements in both worlds (physical world and virtual reality, respectively) were carried out concurrently and independently of each other by two different groups, whereby the graders within the group were also independent of each other. The display of the diameter results at the measurement devices was always blinded to the grader. For the orbital measurements in the physical world, three graders were available, two experienced radiologists and one ophthalmologist. Four additional graders completed the corresponding orbital measurements in the digital world.

### Data acquisition computed tomography

Orbital scans were obtained with the General Electric (GE) computed tomography (CT) system Brightspeed (system number 680542CT0, application software 11BW46.3_SP1-9-1.HP_P_P16_G_GMV, General Electric Company, West Milwaukee, US). The scan protocol consisted of a helical bone skull scanning procedure with a zoom of 145%, left–right angle (L–R) of 0°, and superior-inferior angle (S_I) of − 90°, respectively, and provided 534 images, each with a layer thickness of 0.625 mm.

### Physical world measurement

A total of 6 metal beads (metal bean, diameter 2 mm, Vonarburg AG, Luzern, Switzerland) were randomly fixed to the skull with a good adhesive, widely used and transparent plastic tape (3M, Transpore Surgical Tape, Maplewood, US). Three different diameters per orbit were considered. The diameter directions were measured from the outside border of the beads in the frontal, longitudinal and oblique direction, respectively. Similarly, 3 additional metal pins (Metal Pearl, length of 25 mm, Vonarburg AG, Luzern, Switzerland) were fixed in the mentioned directions, with one axially placed inside the orbit. Before the start of the study, sample CT scanning was performed on both materials to determine their artifact signal characteristics. The positive properties of these materials were then reviewed and approved by the most experienced radiologist (TV).

A commercially available digital precision caliper was used for the diameter measurements in the physical world (DigiMax, Wiha Werkzeuge GmbH, Schonach, Germany). Such a caliper is often used for manufacturing inspection to measure the exact diameter of an object and shows a relatively high measuring accuracy and is easy and fast to operate. The used caliper has a 5-digit digital display with 7.5 mm numeral height for easy reading and margins of error of 0.05 mm. A pair of jaws fixed to a long beam marked with a ruler was used to perform outside (frontal and longitudinal) measurements. Depth measurement were achieved with an included depth probe that slides along the beam which was suitable for the sagittal measurements in this study.

### Digital VR caliper

A VR program (SpectoVR, version 3.1.0); Center for medical Image Analysis & Navigation, University of Basel, Switzerland) was predominantly programmed for an immersive experience and written in C++/OpenGL. This enabled importing of volumetric medical data, in this case CT data. Volume data were transferred from the CT scanner in DICOM format into the VR environment (HTC Vive Pro, Xindian District, New Taipei City, Taiwan) and instantly volume rendered. The ray-casting system was capable of rendering the CT voxel data with dynamic lighting at the native refresh rate of the head mounted display—90 frames per second per eye. The application was deployed on a Windows PC (HP Z640, Intel CPU E5-2620 v4, 32GB DDR4 RAM, NVidia GTX 1080 GPU, Windows 10). A digital VR caliper tool was programmed, which allowed, the user to place two markers in the volume with a motion tracked HTC Vive controller. The markers were connected with a virtual line and the user was allowed to reposition them before saving the final measurement. The positions and the number of adjustments were logged without displaying them to the user during measurement.

In order to exclude a performance bias, the following precautions were taken: The physical and digital instruments were reset to zero before each measurement. All measurements were blinded three times: The two grader groups had no contact with each other, each grader carried out the measurement alone and the display of the measured values was made invisible each time before a measurement was read.

In both worlds the skull was free and not bound to a spine or any object, so the graders allowed the skull to be moved in all directions to allow the best possible and accessible measurement. An explanatory video (Supplementary Video V1) shows the VR measurements to help to better appreciate the measurements.

#### Representation of VR measurements (Supplementary Video V1)

This video demonstrates how a diameter measurement is performed in virtual reality. It is important to realize that in VR the measuring points can be frozen and observed or refined individually until the grader judges his measurement to be ideal. In addition, this refinement can be quantified and objectively compared between the graders to better examine the grader performance.

### Statistical analysis

Independent samples t-test was performed to test whether two samples were different from each other and not due to chance. As the samples showed different variances, a Welch's t-test was added to improve the analysis. Statistical significance was defined at a significance level of 1%. Cohen's d was used to indicate the standardized difference between two means, i.e. to what extent the two distributions differ.

### Bland–Altman plot

The average values for each measuring modality have been considered as paired measurements in the physical and virtual world. The confidence interval has been calculated as where $\overline{d}$ is the mean and $s$ is the standard deviation of the differences.

### Intraclass correlation coefficient (ICC)

Two-way model k raters were selected and subsequently, each subject was measured by the same set of k raters. Single measures, even though more than one measure was taken in the experiment, reliability was applied to a context where a single measure of a single rater will be performed. The reliability was applied to a context where measures of k raters will be averaged for each subject. Agreement of the agreement between two raters is of interest, including systematic errors of both raters and random residual errors. Consistency in the context of repeated measurements by the same rater, systematic errors of the rater are canceled and only the random residual error is kept. The consistency ICC cannot be estimated in the one-way random effects model, as there is no way to separate the inter-rater and residual variances. A 95% confidence interval (CI) was used to define the Intraclass correlation, where values > 0.70 suggest a high level of agreement between the graders^40^.

### William’s index

A similar measure was used with regard to the average angular similarity between rater *i* and *j*:

$$s\left({v}_{i},{v}_{j}\right):=1-\frac{2}{\pi }{cos}^{-1}\left(\frac{{v}_{i}^{T}{v}_{j}}{\Vert {v}_{i}\Vert \Vert {v}_{j}\Vert }\right)$$

where ${v}_{i},{v}_{j}$ are vectors containing the average measurements of the respective rater.

The William’s index can be interpreted as follows: if this index is greater than 1, it can be concluded that rater *j* agrees with the other raters at least as often as they agree with each other. An index greater than 1 is deemed good, whereas an index below 1 is deemed inadequate.

## Supplementary information

Supplementary Video 1.

## Data Availability

The datasets generated and analyzed during the current study are available from the corresponding author on reasonable request.
